# Community Composition of Nitrous Oxide-Related Genes in Salt Marsh Sediments Exposed to Nitrogen Enrichment

**DOI:** 10.3389/fmicb.2018.00170

**Published:** 2018-02-12

**Authors:** John H. Angell, Xuefeng Peng, Qixing Ji, Ian Craick, Amal Jayakumar, Patrick J. Kearns, Bess B. Ward, Jennifer L. Bowen

**Affiliations:** ^1^Biology Department, University of Massachusetts Boston, Boston, MA, United States; ^2^Department of Geosciences, Princeton University, Princeton, NJ, United States

**Keywords:** salt marsh, nitrous oxide, nutrient enrichment, denitrification, *norB*, *nosZ*

## Abstract

Salt marshes provide many key ecosystem services that have tremendous ecological and economic value. One critical service is the removal of fixed nitrogen from coastal waters, which limits the negative effects of eutrophication resulting from increased nutrient supply. Nutrient enrichment of salt marsh sediments results in higher rates of nitrogen cycling and, commonly, a concurrent increase in the flux of nitrous oxide, an important greenhouse gas. Little is known, however, regarding controls on the microbial communities that contribute to nitrous oxide fluxes in marsh sediments. To address this disconnect, we generated profiles of microbial communities and communities of micro-organisms containing specific nitrogen cycling genes that encode several enzymes (*amoA, norB, nosZ)* related to nitrous oxide flux from salt marsh sediments. We hypothesized that communities of microbes responsible for nitrogen transformations will be structured by nitrogen availability. Taxa that respond positively to high nitrogen inputs may be responsible for the elevated rates of nitrogen cycling processes measured in fertilized sediments. Our data show that, with the exception of ammonia-oxidizing archaea, the community composition of organisms involved in the production and consumption of nitrous oxide was altered under nutrient enrichment. These results suggest that previously measured rates of nitrous oxide production and consumption are likely the result of changes in community structure, not simply changes in microbial activity.

## Introduction

Salt marshes provide numerous ecosystem services (Deegan, 1993; Chmura et al., 2003; Gedan et al., 2011) including the removal of fixed nitrogen (N) from the environment (Valiela and Teal, 1979). This service acts to limit eutrophication (Valiela and Cole, 2002), which is crucial for the maintenance of healthy coastal waters (Deegan et al., 2012), and will grow in importance as nitrogen loading to the coast increases in the future (Galloway et al., 2004; Canfield et al., 2010). The location of salt marshes at the interface of the land and sea allows them to intercept and filter nutrient-laden water as it leaves terrestrial landscapes (Howes et al., 1996; Mitsch et al., 2005; Drake et al., 2009; Brin et al., 2010). In marsh sediments, a portion of N is sequestered in organic rich peat. The vast majority, however, is removed through microbial N-cycling processes, in particular denitrification (Anderson et al., 1997; Tobias et al., 2001; Hamersley and Howes, 2005), which can account for up to 84% of fixed nitrogen removal in marsh systems (Valiela and Teal, 1979).

Denitrification is the stepwise reduction of nitrate (NO_3_^-^) to a nitrogenous gas, either nitrous oxide (N_2_O) or dinitrogen gas (N_2_), generally under anoxic conditions (Zumft, 1997). The last two steps of complete denitrification are the reduction of nitric oxide (NO) to N_2_O, followed by the reduction of N_2_O to N_2_. These steps are mediated by the enzymes nitric oxide reductase (NOR) and nitrous oxide reductase (N_2_OR), which are frequently assessed in the environment by examination of the *norB* and *nosZ* genes, respectively (Dalsgaard et al., 2014; Kearns et al., 2015). Canonical denitrifiers contain the entire suite of enzymes necessary for the complete reduction of NO_3_^-^ to N_2_. However, the denitrification pathway also shows a remarkable degree of modularity (Graf et al., 2014; Roco et al., 2017), with many known organisms containing a subset, or even just a single enzyme within the pathway. Modularity implies that individual N transformations may be mediated by distinct communities of microbes, which may respond to environmental conditions differently. If changing environmental conditions alter the production but not the consumption of N_2_O, the benefit of N removal from the marsh might be offset by the production of this important greenhouse gas.

In addition to denitrification, salt marsh sediments are hotspots for other processes in the N cycle (Kaplan et al., 1979; Valiela and Teal, 1979). Of particular importance is nitrification (Dollhopf et al., 2005), the two-step aerobic oxidation of ammonia (NH_3_) to NO_3_^-^, through the intermediate nitrite (NO_2_^-^). Nitrification supplies the NO_3_^-^ that is needed for denitrification, which is typically limiting in oxygen-depleted sediments. This linkage, referred to as coupled nitrification–denitrification, often represents the largest N-loss process from salt marshes (Patrick and Reddy, 1976; White and Howes, 1994). The first step of nitrification (NH_4_^+^ oxidation to NO_2_^-^) is catalyzed by the enzyme ammonia monooxygenase, which is encoded by the *amo* gene. This gene is present in bacteria and archaea (Kowalchuk and Stephen, 2001; Leininger et al., 2006). Additionally, some nitrifying bacteria contain *nor* and can denitrify (reducing NO_2_^-^ to N_2_O) in a process called nitrifier-denitrification (Wrage et al., 2001; Kool et al., 2010). Nitrifer-denitrifier *nor*, though similar in function, is genetically distinct from canonical denitrifier *nor* and can be differentiated in molecular analyses (Casciotti and Ward, 2005).

Nitrous oxide, a product of N-cycling processes, is a potent greenhouse gas with approximately 300 times the warming potential of carbon dioxide [Environmental Protection Agency (EPA), 2013], thereby having a significant effect on global warming (Joos and Spahni, 2008). Additionally, N_2_O is predicted to become the dominant ozone-depleting substance this century (Ravishankara et al., 2009). During the oxidation of ammonia, N_2_O can be produced as a by-product from hydroxylamine decomposition in ammonia-oxidizing bacteria (Poth and Focht, 1985), and via a nitric oxide intermediate in ammonia-oxidizing archaea, as well as via abiotic processes (Kozlowski et al., 2016). In denitrification, N_2_O is an integral intermediate in the stepwise process. However, some portion of the denitrified N “leaks” out as N_2_O, as described in the hole-in-the-pipe hypothesis (Firestone and Davidson, 1989). Some of this leaked N_2_O may be the result of denitrifiers that lack the *nosZ* gene and whose denitrification pathway terminates at N_2_O production (Philippot et al., 2011). Conversely, two clades of bacteria contain *nosZ* genes that are phylogenetically distinct from the canonical denitrifier *nosZ*, termed atypical *nosZ*. These organisms can scavenge free N_2_O from the environment, possibly making them important N_2_O sinks in salt marsh sediments (Sanford et al., 2012; Jones et al., 2013). While nitrification and denitrification represent important sources and sinks for N_2_O, other processes, including dissimilatory nitrate reduction to ammonium (DNRA) also likely produce N_2_O (Sun et al., 2016), though their contribution to overall N_2_O production is poorly characterized.

Geochemical studies have shown that the magnitude of N_2_O fluxes, and the relative contribution of denitrification and nitrification to that flux, are controlled by a combination of oxygen availability (Anderson et al., 1993; Khalil et al., 2004), soil moisture content (Klemedtsson et al., 1988; Bateman and Baggs, 2005), nitrogen load (Smith et al., 1998; Davidson et al., 2000), and carbon content (Swerts et al., 1996). Prior work suggests that nitrification dominates N_2_O production in areas with low soil moisture and high oxygen availability, while denitrification is the main source of N_2_O in wet, anoxic conditions, with nitrogen content controlling the magnitude of the flux (Moseman-Valtierra et al., 2011, 2015).

The experimental fertilization plots established in the Great Sippewissett Salt Marsh (Valiela et al., 1973) on Cape Cod, MA, United States provide an ideal site to identify how microbes in general, and specifically microbes involved in N_2_O fluxes, respond to changes in environmental conditions. Salt marsh ecosystems have several habitats that provide natural gradients in elevation, degree of saturation of the soils (Bertness and Ellison, 1987; Pennings et al., 2005), and oxygen (Teal and Kanwisher, 1961; Maricle and Lee, 2002). The experimental plots have also received nitrogen additions since the early 1970s (Valiela et al., 1973; Kaplan et al., 1979; Hamersley and Howes, 2005; Ji et al., 2015; Peng et al., 2016) thereby allowing us to assess the effects of nutrient supply on genes involved in the nitrogen cycle. Understanding the environmental controls on N_2_O fluxes depends on identifying the microbes that are ultimately responsible for the production and consumption of N_2_O in salt marsh sediments. How the microbial communities that mediate these processes respond to changes in environmental conditions, however, is largely unknown.

We used high-throughput sequencing and a functional gene microarray analysis to examine changes in the microbial community and specific N cycle genes in the Great Sippewissett Marsh plots. We hypothesized that the community composition of nitrogen cycling genes involved in N_2_O production and consumption (*amoA*, *norB*, and *nosZ*) would vary as a function of fertilization, habitat, and depth. Further, we hypothesized that the patterns of variation in these genes, when compared to previously published fluxes of N_2_O from the marsh surface, would allow us to infer which groups of microbes (nitrifiers, denitrifiers, or nitrifier-denitrifiers) were likely responsible for the N_2_O flux. We predicted that populations of ammonia oxidizers would vary as a function of NH_4_^+^ concentration and O_2_ availability and that denitrifiers would vary as a function of NO_3_^-^ concentration and O_2_ availability. Finally, as has been seen in this and other marsh fertilization experiments (Bowen et al., 2011; Kearns et al., 2016), we hypothesized that the overall microbial community, which includes both active and dormant microbes, would not vary as a function of fertilization, but the potentially active community would show a shift in community structure as nutrient enrichment increases.

## Materials and Methods

### Field Sampling

We collected samples in August 2012 from the experimental long-term fertilization plots at Great Sippewissett Salt Marsh, Falmouth, MA, United States [41° 35′ 3.1″ N, 70° 38′ 17.0″ W] (Valiela et al., 1973). During low tide, we took sediment cores using sterile 30 ml cut-off syringes from two control plots (C) which do not receive fertilization, two highly fertilized (HF, 2.52 g N m^-2^ week^-1^) plots, and two extra highly fertilized (XF, 7.56 g N m^-2^ week^-1^) plots. In each of the duplicated plots, we took duplicate cores in low marsh and high marsh habitats, for a total of four cores per plot (*n* = 6 plots). From each core we took sediment samples from the surface and depth for nucleic acid extraction and analysis, resulting in eight total samples per plot (2 depths × 2 habitats × 2 duplicates). In all plots sampled, the low marsh habitat consisted of monocultures of the tall ecotype of *Spartina alterniflora*. High marsh habitats in C and HF plots consisted of monocultures of the short ecotype of *Spartina alterniflora*, but in the XF plots, high marsh habitats were dominated by *Distichlis spicata*. Sediment cores were immediately frozen in liquid nitrogen, stored on dry ice for transport back to the lab, and kept frozen at -80°C until processed.

Oxygen (O_2_) measurements were made in the field using a Clark-type microelectrode (OX-500, Unisense^TM^, Aarhus, Denmark) coupled with a Unisense^TM^ micrometer and micromanipulator. The probe was two-point calibrated in the field following manufacturer’s instructions. Sediment O_2_ profiles were generated by taking measurements at 500 μm increments to a depth of 3 cm. In each experimental plot, we took one profile in the low marsh habitat and a second profile in the high marsh habitat. We removed any measurements taken above the sediment surface from the dataset, which was then normalized by setting the lowest reproducible value as 0% oxygen and setting any remaining negative values to a value of zero.

### Geochemical Analysis

Nutrient concentrations, sediment properties, and geochemical rates were measured from sediment cores taken directly adjacent to cores used for molecular analysis. Sediment moisture content was measured gravimetrically by comparing weights of sediment before and after drying to a constant weight at 65°C. As described in detail in Peng et al. (2016), nutrients (NO_3_^-^, NH_4_^+^) in pore water were extracted by potassium chloride; NO_3_^-^ concentrations were measured chemiluminescently using a NO/NOx Analyzer (Model 200E, Teledyne^TM^, Thousand Oaks, CA, United States) with a hot (90°C) acidified vanadium (III) reduction column (Garside, 1982; Braman and Hendrix, 1989), and NH_4_^+^ concentrations were measured colorometrically (Strickland and Parsons, 1968) on a UV-Visible Spectrophotometer (UV-1800, Shimadzu^TM^, Kyoto, Japan). The limit of detection for both analyses was 0.5 μM. Statistical differences among treatments were assessed using analysis of variance (ANOVA) followed by Tukey *post hoc* tests to test specific comparisons. *t*-Tests were used to test for differences in pairwise comparisons between high and low marsh samples and between shallow and deep samples.

### Nucleic Acid Extraction

Sediment cores were sectioned, while frozen at -20°C, using a microtome at 1 mm increments to a depth of 3 cm. During the sectioning process, sediment slices were collected on paraffin wax film (Parafilm M, Bemis, Oshkosh, WI, United States) before being transferred to sterile microcentrifuge tubes. Between each section, the paraffin film was changed and the microtome blade and spatulas used for transferring the sediment to the tubes were cleaned with 70% ethanol to minimize contamination among samples. DNA and RNA were co-extracted from the topmost section (0–1 mm; “shallow”) and the bottommost section (29–30 mm; “deep”) of each core (*n* = 48) for comparison between the redox extremes found in the top 3 cm of marsh sediment. Nucleic acids were co-extracted using a MoBio^TM^ PowerSoil^®^ RNA Isolation Kit (MoBio^TM^, Carlsbad, CA, United States) with a DNA Isolation Accessory Kit according to the manufacturer’s instructions. RNA extracts were checked for DNA contamination via polymerase chain reaction (PCR) using universal 16S rRNA gene primers 515F and 806R (Bates et al., 2011; Caporaso et al., 2011). Samples with DNA contamination were treated with DNase I (New England Biolabs^TM^, Ipswich, MA, United States). Complementary DNA (cDNA) synthesis was performed using Invitrogen^TM^ Superscript III^®^ First-Strand Synthesis System for RT-PCR (Thermo Fisher Scientific^TM^, Cambridge, MA, United States) with random hexamers according to the manufacturer’s instructions. Nucleic acid concentrations from each sample were measured on a Qubit^®^ 2.0 fluorometer (Thermo Fisher Scientific^TM^, Cambridge, MA, United States).

### Microarray Analysis

We used glass microarray slides (DeRisi et al., 1997) containing probes for several key nitrogen-cycling genes including *norB*, *nosZ*, and archaeal, but not bacterial, *amoA* (array name BC016). Probe sets were designed using previously described algorithms (Ward et al., 2007; Bulow et al., 2008). Ninety-nine *amoA* probes were identified from published sequences (Biller et al., 2012). Forty-three *norB* and 71 *nosZ* probes were also identified using published sequences in addition to sequences obtained from clone libraries made using DNA extracted from our sampling locations in the Sippewissett plots (Kearns et al., 2015). Forty-three additional *nosZ* probes, labeled as “WNZ” or “WnosZ2” were included to capture both clades of atypical *nosZ* sequences, atypical *nosZ1* and atypical *nosZ2*, respectively. Each probe is designed to hybridize to all sequences within 87 ± 3% identity of the 70-mer probe sequence. We refer to the sequences that hybridize to a particular probe as an archetype; representing a group of related sequences (Taroncher-Oldenburg et al., 2003).

Two fertilization levels were used for microarray analysis. DNA samples from shallow and deep sediments from high and low marsh habitats in C and XF plots were used for microarray analysis as previously described (Ward and Bouskill, 2011). Briefly, 50 ng of DNA from each sample was digested with Hinf1 for 2 h followed by ethanol precipitation. Digested DNA was used for labeling with a BioPrime^®^ kit (Thermo Fisher Scientific^TM^, Cambridge, MA, United States) using random primers and a custom 1.2 nM dNTP mix with dUaa, followed by ethanol precipitation. The precipitated DNA was dissolved in 4.5 μl of 100 nM NaCO_3_ (pH 9) before the addition of 4.5 μl of Cy3 dye and left to incubate overnight. Samples were then purified using a QIAquick^®^ PCR cleanup kit (Qiagen^TM^, Valencia, CA, United States) as previously described (Ward and Bouskill, 2011). DNA concentration of the targets was measured on a Qubit^®^ fluorometer and the volume required for 1000 ng of DNA was aliquoted into two separate tubes per sample, dried down in a speedvac, and stored frozen until processed.

Microarrays were hybridized overnight in an ozone-free room and washed three times (Ward and Bouskill, 2011) before scanning on an Agilent^TM^ laser scanner 4300 (Agilent^TM^, Palo Alto, CA, United States). Microarray images were analyzed using GenePix^®^ 6.0 software. Relative fluorescence ratio (RFR), the percent that each probe contributes to the total fluorescence of the probe group, was used for statistical analysis. Replicate microarrays were hybridized and replicate features on the same array were averaged to calculate the RFR for each probe. Statistical analysis of microarray data was done in R (R Development Core Team, 2008), including generation of non-metric multidimensional scaling (NMDS) plots for each probe group calculated using Bray–Curtis similarities in the vegan package (Oksanen et al., 2015). Also in vegan, we used adonis (Anderson, 2001), a method that uses distance matrices of Bray–Curtis similarity values for permutational multivariate analyses of variance (PERMANOVA), to test for significant differences in gene composition that resulted from fertilization. Probes that differed significantly among treatment, habitat, or depth were determined via Kruskal–Wallis test in R. Significance was assessed at an alpha of 0.05. The microarray data from BC016 are available via the Gene Expression Omnibus under GEO Accession GSE108888.

### Sequencing

Samples from one plot at each level of treatment (C, HF, XF) were used for sequencing the 16S rRNA gene and its gene product, 16S rRNA, to determine the community composition of the total and the potentially active microbial taxa, respectively. While there are limitations to using 16S rRNA as a proxy for microbial growth (Blazewicz et al., 2013), 16S rRNA provides a snapshot of which taxa may potentially be active in the microbial community. Samples (*n* = 48) included 16S rRNA and the 16S rRNA gene from two depths and two habitats from each of the three plots. The V4 region of 16S rRNA and the 16S rRNA gene were amplified via PCR using barcoded primers 515F and 806R with Illumina^TM^ adaptors (Caporaso et al., 2011, 2012). Amplicons were generated in triplicate reactions, pooled, and gel-purified using a Qiagen^TM^ QIAquick^®^ Gel Extraction Kit (Qiagen^TM^, Valencia, CA, United States) according to the manufacturer’s instructions. The concentration of purified amplicons was measured on a Qubit^®^ fluorometer and samples were pooled in equal concentrations and sequenced on an Illumina^TM^ MiSeq^®^ (Illumina^TM^, San Diego, CA, United States) using paired-end V2 300 cycle chemistry.

Sequences were quality filtered and analyzed in QIIME (Caporaso et al., 2010) and R (R Development Core Team, 2008). All quality-filtered sequences are available in the Sequence Read Archive (Accession No.: PRJNA423244). Paired-end reads were joined with fastq-join (Aronesty, 2013) and quality filtered in QIIME following the protocols of Bokulich et al. (2013). Sequence data were checked for chimeras using UCHIME (Edgar et al., 2011) in *de novo* mode. Swarm (Mahé et al., 2014) was used to pick operational taxonomic units (OTUs) using 97% sequence identity, with taxonomy assigned using UCLUST (Edgar, 2010) and Greengenes (version 13.5) as a reference database. Sequence data were further quality filtered to exclude OTUs that were only present once in the dataset or that could not be assigned taxonomy within the Bacterial Kingdom. Chimeras and those sequences that could not be assigned taxonomy represented less than 3% of the dataset. We rarefied sequence data in QIIME to a depth of 9500 sequences per sample for 16S rRNA genes and to a depth of 7000 sequences per sample for 16S rRNA. Taxonomy tables were generated in QIIME and exported to R (R Development Core Team, 2008) for analysis. R was used to generate NMDS plots of Bray–Curtis similarities using the vegan package. Statistical differences in community composition were calculated in R using adonis on Bray–Curtis similarity values.

## Results

### Environmental Conditions

**FIGURE 1:**
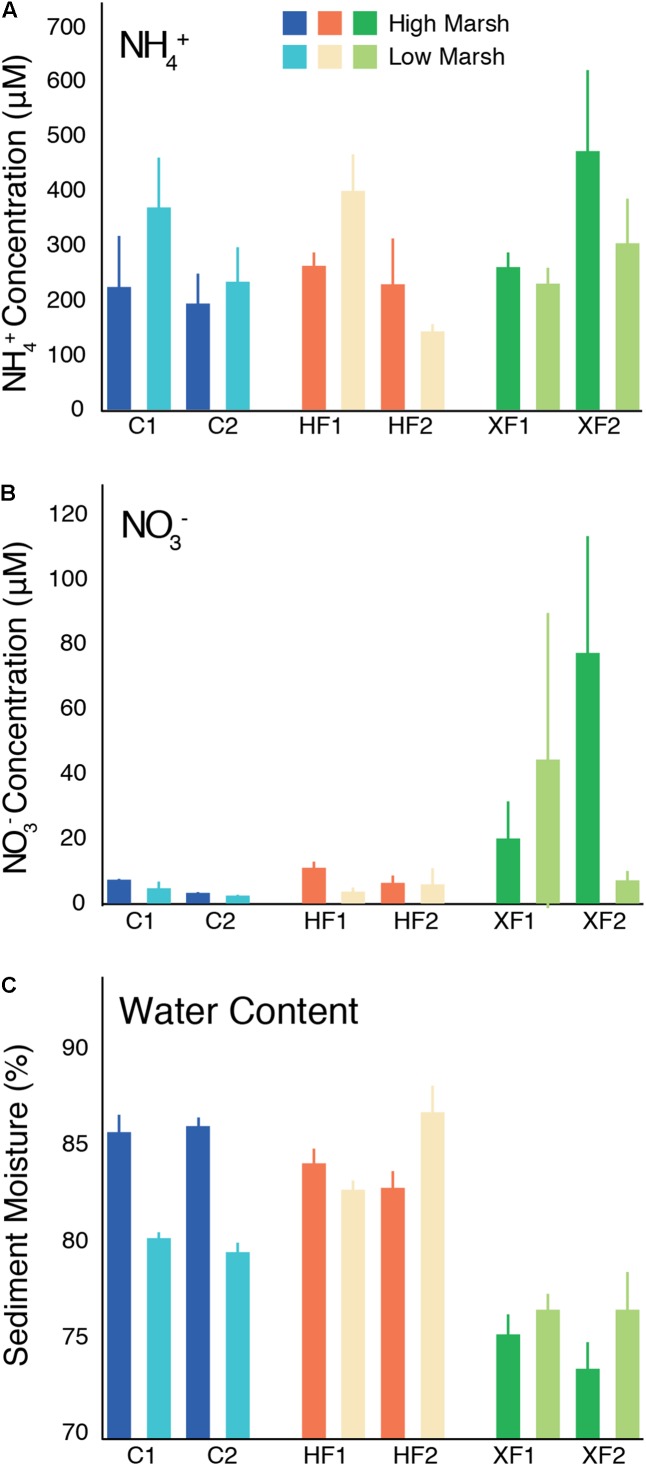
Environmental data from sediments collected from the high and low marsh habitats of two replicate control (C), highly fertilized (HF) and extra highly fertilized (XF) salt marsh plots. Data include **(A)** pore-water ammonium (NH_4_^+^) concentrations (μM), **(B)** pore-water nitrate (NO_3_^-^) concentrations (μM), and **(C)** sediment moisture content (%). Each bar represents the average (and range) of values from two replicate cores.

In the Sippewissett Marsh plots, pore water concentrations of NH_4_^+^ were variable and averaged between 137 and 467 μM (**Figure 1A**). While NH_4_^+^ concentrations varied considerably among samples, they did not systematically differ as a function of habitat or treatment (ANOVA, *F* = 1.179, *p* = 0.321). In contrast, pore water NO_3_^-^ concentrations (up to 80 μM) did vary as a function of treatment (ANOVA, *F* = 7.609, *p* = 0.002), with XF plots containing significantly more NO_3_^-^ than HF (Tukey HSD, *p* = 0.007) and C (*p* = 0.005) plots (**Figure 1B**). There was no significant difference between C and HF pore water NO_3_^-^ concentrations (*p* = 0.966). Moisture content also varied as a function of treatment (ANOVA, *F* = 39.57, *p* < 0.001; **Figure 1C**) as XF plots were significantly drier than HF (Tukey HSD, *p* < 0.001) and C (*p* < 0.001) plots. HF plots had the wettest sediments and contained significantly more water than C plots (*p* = 0.011), driven primarily by the higher water content of the low marsh in HF plots compared to C plots. Habitat type also affected moisture content. Low marsh habitats had significantly drier sediments than high marsh habitats in C plots (*t*-test, *t* = 5.40, *p* < 0.001), but significantly wetter sediments in HF plots (*t*-test, *t* = -2.78, *p* = 0.010) and XF plots (*t*-test, *t* = -4.53, *p* < 0.001).

**FIGURE 2:**
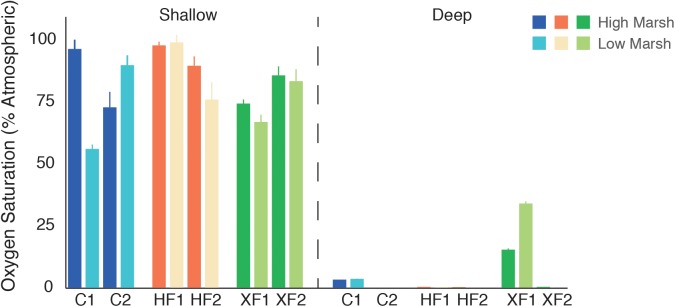
Average oxygen content (% saturation) of measurements taken within the top millimeter of surface sediment (shallow) and sediment collected from 29 to 30 mm depth (deep) for each experimental core.

Oxygen content in shallow and deep samples was calculated by averaging the O_2_ values for the top millimeter (shallow) and bottom millimeter (deep) of the oxygen profile (**Figure 2**). Oxygen availability varied with depth, as surface sediments contained significantly more oxygen than deep sediments (*t*-test, *t* = 11.27, *p* < 0.001). Only a single XF plot had detectable amounts of oxygen at depth. Oxygen values differed among samples, but there was no overall effect of treatment or habitat on oxygen availability. Peng et al. (2016) published more detailed oxygen profiles from this system.

### Functional Gene Analysis

**FIGURE 3:**
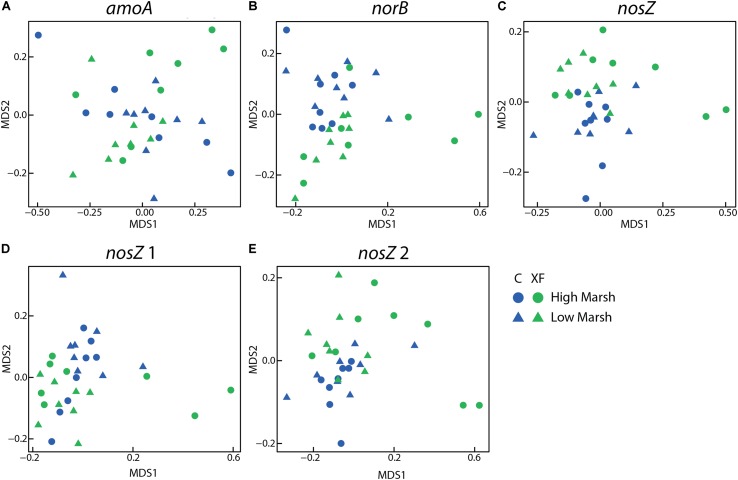
Non-metric multidimensional scaling (NMDS) plots of RFR microarray data for AOA *amoA* **(A)**, *norB* **(B)**, *nosZ* **(C)**, atypical *nosZ* 1 **(D)**, and atypical *nosZ* 2 **(E)**. All but AOA *amoA* differ significantly by treatment using adonis. None differ significantly by habitat or depth. Each figure includes eight samples (2 depths × 2 habitats × 2 duplicate cores) per plot and there were four plots (two control and two XF-fertilized plots).

Non-metric multidimensional scaling plots of Bray–Curtis similarity for each probe group on the microarray indicated that the community structure of most genes responsible for N_2_O production and consumption were significantly different between fertilized and unfertilized plots (**Figure 3**). The only gene that did not differ by fertilization treatment was the *amoA* gene from ammonia-oxidizing archaea. Nitrogen cycling genes that varied significantly by fertilization level include *norB* (adonis, *F* = 4.59, *p* = 0.001, *R*^2^ = 0.13), *nosZ* (*F* = 3.52, *p* = 0.009, *R*^2^ = 0.11), atypical *nosZ* 1 (*F* = 3.89, *p* = 0.012, *R*^2^ = 0.11), and atypical *nosZ* 2 (*F* = 3.74, *p* = 0.030, *R*^2^ = 0.11). While these differences in community structure were significant, the amount of variation explained by nutrient enrichment was fairly low, with no *R*^2^ value greater than 0.15. Community structure of nitrogen-cycling genes did not vary significantly by depth or habitat.

To identify which archetypes, related sequences that hybridize to a particular probe, were important in explaining differences in community structure of key N-cycle genes, we identified archetypes that accounted for greater than 1% of the total fluorescence for their probe set (RFR > 0.01) and that were significantly different as a result of fertilization and/or habitat using a Kruskal–Wallis test (**Table 1**). Of the archetypes that differed significantly, nearly all varied as a function of fertilization or a combination of fertilization and habitat. Only one archetype, nosZ23, a low-signal probe that was derived from salt marsh clone libraries (Kearns et al., 2015), differed solely as a function of habitat. Additionally, the *norB*, *nosZ*, and atypical *nosZ1* archetypes with the highest RFR for the gene, indicating the highest relative abundance in their community, all varied by treatment. Surprisingly, three archetypes, *norB39*, *nosZ64*, and *WnosZ16*, had a significantly higher relative hybridization in unfertilized sediments. The majority of the remaining archetypes that varied significantly as a function of fertilization, especially for *nosZ*, were relatively more abundant in fertilized sediments (**Table 1**). Archetypes that varied significantly by fertilization accounted for only 8.1% of the *amoA* community, but accounted for 41.1% for *norB*, 25.9% for *nosZ*, 43.7% for atypical *nosZ* 1, and 22.5% for atypical *nosZ* 2 communities (**Table 1**). Many of these archetypes are only distantly related to cultured microbes and were originally derived from sequences identified in Sippewissett Marsh samples.

**Table 1:** Closest cultured BLAST match, percent identity, and the average relative fluorescence ratio (RFR) per habitat for each archetype that accounted for greater than 1% of the total fluorescence and was significantly different among treatments by Kruskal–Wallis H-test and a significance threshold of *p* < 0.05.

Probe	Closest cultured BLAST match	% Identity	RFR in C plots	RFR in XF plots
AOA26	*Candidatus Nitrososphaera evergladensis*	87	**0.029 (0.009)**	0.025 (0.013)
AOA22	*Candidatus Nitrosotenuis* sp.	89	0.014 (0.006)	**0.022 (0.012)**
AOA50	*Candidatus Nitrosopumilus* sp.	81	0.012 (0.004)	**0.017 (0.006)**
AOA4	*Enterobius vermicularis*	94	0.009 (0.004)	**0.015 (0.008)**
AOA70	*Nitrosopumilus maritimus*	84	0.009 (0.004)	**0.014 (0.004)**
NorB39	*Thioalkalivibrio paradoxus*	100	**0.184 (0.038)**	0.136 (0.04)
NorB34	*Hahella chejuensis*	100	**0.063 (0.017)**	0.055 (0.038)
NorB2	*Azoarcus* sp.	100	0.029 (0.008)	**0.049 (0.008)**
NorB11	*Magnetospirillum gryphiswaldense*^∗^	84	**0.031 (0.008)**	0.029 (0.007)
NorB14	*Nitrosococcus oceani*	100	0.023 (0.009)	**0.031 (0.014)**
NorB8	*Alkalilimnicola ehrlichii*^∗^	89	0.02 (0.005)	**0.025 (0.007)**
NorB15	*Nitrosospira briensis*	100	**0.021 (0.005)**	0.015 (0.005)
NorB41	*Ruegeria mobilis*	88	**0.016 (0.01)**	0.014 (0.005)
NorB29	*Magnetospirillum gryphiswaldense*	85	0.012 (0.006)	**0.018 (0.008)**
NorB27	*Nitrosospira* sp.	100	0.013 (0.004)	**0.016 (0.007)**
NorB5	*Dinoroseobacter shibae*^∗^	90	**0.015 (0.005)**	0.014 (0.008)
NosZ64	*Rhodanobacter denitrificans*	100	**0.109 (0.022)**	0.089 (0.043)
NosZ61	*Rhodospirillum centenum*	100	0.023 (0.003)	**0.028 (0.005)**
NosZ48	*Paracoccus* sp.^∗^	83	0.02 (0.005)	**0.024 (0.004)**
NosZ30	*Paracoccus* sp.^∗^	88	0.019 (0.004)	**0.022 (0.004)**
NosZ35	*Achromobacter cycloclastes*	100	**0.024 (0.007)**	0.016 (0.006)
NosZ32	*Thalassospira xiamenensis*^∗^	81	0.015 (0.005)	**0.023 (0.01)**
NosZ29	*Ruegeria pomeroyi*^∗^	86	**0.022 (0.008)**	0.015 (0.006)
NosZ1	*Mesorhizobium* sp.^∗^	77	0.016 (0.003)	**0.02 (0.003)**
NosZ2	*Hoeflea* sp.^∗^	89	**0.02 (0.005)**	0.015 (0.005)
WNZ16	*Anaeromyxobacter dehalogenans*	83	**0.182 (0.054)**	0.144 (0.081)
WNZ1	*Roseateles depolymerans*	72	0.061 (0.016)	**0.083 (0.022)**
WNZ13	*Streptomyces raramycinicus*	88	0.055 (0.011)	**0.064 (0.009)**
WNZ20	*Anaeromyxobacter* sp.	81	0.035 (0.007)	**0.048 (0.007)**
WNZ19	*Burkholderia* sp.	83	0.033 (0.006)	**0.048 (0.006)**
WNZ25	*Burkholderia ambifaria*	96	0.025 (0.006)	**0.032 (0.005)**
WnosZ2_1	*Anaeromyxobacter dehalogenans*	100	**0.11 (0.026)**	0.077 (0.024)
WnosZ2_15	*Rhodothermus marinus*	84	0.045 (0.012)	**0.064 (0.011)**
WnosZ2_11	*Salinibacter ruber*	100	0.019 (0.005)	**0.024 (0.005)**
WnosZ2_8	*Rubrivivax gelatino*	100	**0.022 (0.008)**	0.019 (0.004)
WnosZ2_4	*Desulfomonile tiedjei*	100	0.018 (0.004)	**0.023 (0.004)**
WnosZ2_13	*Gemmatimonas aurantiaca*	84	0.013 (0.003)	**0.016 (0.004)**


*Bold text indicates the treatment with the highest mean ( ± SD) fluorescence (*n* = 8). ^∗^Archetypes derived from marsh sequences (Kearns et al., 2015) that have only distant relationships to known taxa. Note that only ammonia-oxidizing archaea were present on the microarray, not ammonia oxidizing bacteria. We assessed the importance of ammonia-oxidizing bacteria via analysis of the 16S rRNA gene.*

Surprisingly, depth and the redox conditions associated with depth, were not important in structuring nitrogen-cycling communities, when examined via pairwise comparison of surface to deep samples. Of the archetypes that accounted for greater than 1% of fluorescence in their respective probe sets, only four differed significantly between surface and deep samples, including three *amoA* archetypes; AOA22 (Kruskal–Wallis test, *H* = 3.99, *p* = 0.046), AOA20 (*H* = 7.16, *p* = 0.007), and AOA47 (*H* = 8.64, *p* = 0.003), which most closely resemble Candidatus *Nitrosotenuis* sp., *Nitrososphaera viennensis*, and *Nitrosopumilus maritimus*, respectively, and a single *norB* archetype, *NorB6* (*H* = 4.14, *p* = 0.042), which was derived from the *norB* sequence from *Paracoccus*. These archetypes accounted for only 5% of the hybridization signal of both the *amoA* and *norB* community. No *nosZ* archetypes varied as a function of depth.

### Microbial Community Composition

**FIGURE 4:**
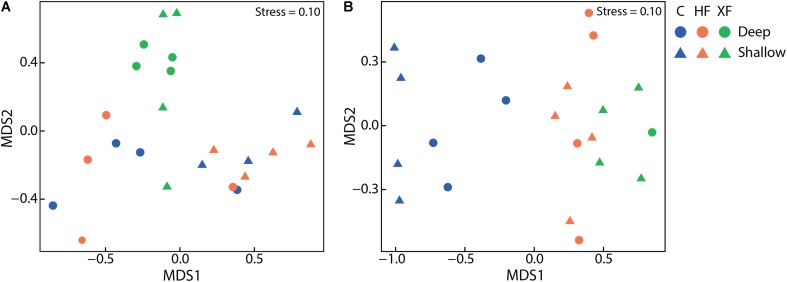
Non-metric multidimensional scaling plots of **(A)** total microbial communities as assessed from sequences of the 16S rRNA gene and **(B)** potentially active communities, as assessed with 16S rRNA, using Bray–Curtis similarities. Total microbial communities varied as a function of treatment and depth and potentially active communities varied solely as a function of treatment.

**FIGURE 5:**
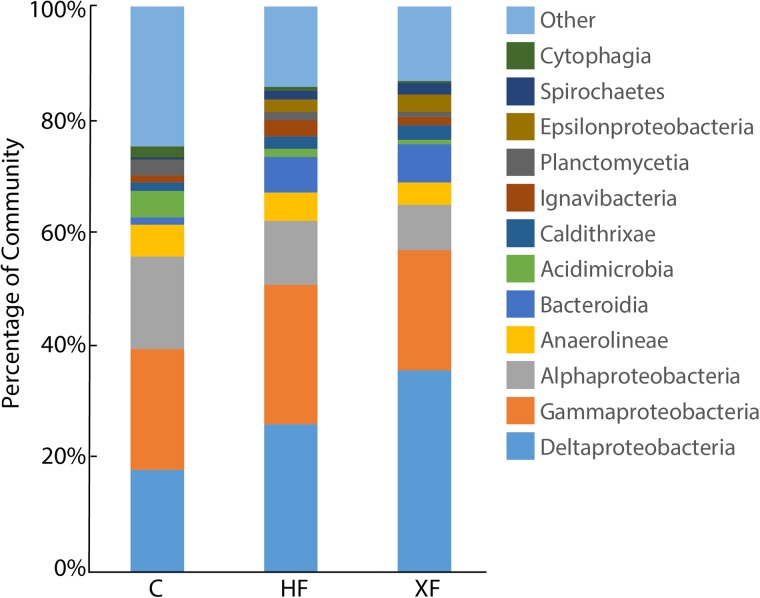
Stacked bar plot of potentially active microbial classes in control (C), highly fertilized (HF), and extra highly fertilized (XF) setiments. Classes identified accounted for at least 1% of the 16S rRNA sequence dataset. Microbial classes that individually accounted for less than 1% of the dataset are combined into the “Other” category.

The structure of the entire microbial community, based on sequence analysis of the 16S rRNA gene, varied as a function of both fertilization (**Figure 4A**; adonis, *F* = 1.63, *p* = 0.005) and depth (adonis, *F* = 1.83, *p* = 0.021). Analysis of 16S rRNA, which, with caveats (Blazewicz et al., 2013), can be used as an indicator of taxa that are potentially active, indicated that fertilization was also important in structuring the active microbial taxa (**Figure 4B**; adonis, *F* = 3.29 *p* = 0.001). Neither habitat nor depth were significant factors in structuring potentially active communities. In the potentially active community, there were 12 bacterial classes that each accounted for greater than 1% of the dataset (**Figure 5**). These dominant classes accounted for approximately 75% of the C, 85% of the HF, and 86% of the XF active microbial communities (**Table 2**).

**Table 2:** Bacterial classes that each accounted for >1% of the potentially active community (based on 16S rRNA), the percentage of the community they represent in fertilized (XF and HF) and control communities, and their sum counts in the rarified dataset.

Bacterial class	Fertilized % (*SD*)	Control % (*SD*)	Count
*Deltaproteobacteria*	27.71 (6.4)	17.02 (3.5)	34745
*Gammaproteobacteria*	21.84 (9.0)	20.24 (3.3)	31208
*Alphaproteobacteria*	9.37 (2.2)	15.56 (2.3)	17235
*Anaerolineae*	4.32 (1.4)	5.32 (1.2)	6909
*Bacteroidia*	6.10 (1.7)	1.25 (0.8)	6253
*Acidimicrobia*	1.10 (0.8)	4.38 (3.7)	3454
*Caldithrixae*	2.19 (1.2)	1.49 (1.1)	2830
*Ignavibacteria*	2.14 (1.3)	1.19 (0.7)	2608
*Planctomycetia*	1.17 (0.6)	2.56 (0.9)	2500
*Epsilonproteobacteria*	2.35 (2.1)	0.10 (0.2)	2196
*Spirochaetes*	1.67 (0.7)	0.36 (0.2)	1721
*Cytophagia*	0.52 (0.3)	1.18 (0.7)	1480


Ammonia-oxidizing archaea were represented on the microarray, but ammonia-oxidizing bacteria (AOB) were not. We can, however, assess the relative abundance of AOB via the sequence data because they form a largely monophyletic clade that can be identified via 16S rRNA gene analysis. Three genera of ammonia-oxidizing bacteria were present in either the total microbial community or the potentially active community (**Table 3**). Of these genera, only *Nitrosomonas* differed significantly by treatment in the potentially active community (Kruskal–Wallis test, *H* = 7.36, *p* = 0.025), where it was more abundant in C samples compared to XF.

**Table 3:** Average counts of three genera of AOB present in the 16S rRNA gene and 16S rRNA sequencing from each habitat.

	C Low	C High	HF Low	HF High	XF Low	XF High
**16S rRNA gene**						
*Nitrosococcus*	0	0	0	0	0	0
*Nitrosomonas*	7.0 (9.9)	46 (53.8)	7.5 (13)	3.5 (3.2)	3.5 (1.8)	4.3 (3.1)
*Nitrosospira*	0	0.3 (0.4)	0.3 (0.4)	0.3 (0.4)	0	0
**16 rRNA**						
*Nitrosococcus*	0	0.3 (0.4)	0	0	0	0
*Nitrosomonas*	28 (34.4)	80.7 (112.2)	2.2 (2.3)	5.3 (9.1)	0	2 (1.6)
*Nitrosospira*	0.5 (0.9)	1.3 (1.6)	0	0.3 (0.4)	0	0


*Standard deviations are in parentheses (*n* = 4). Note different rarefaction depths for DNA (9500 sequences per sample) and RNA (7000 sequences per sample). Numbers < 1 indicate that the taxon was found only in a subset of the replicates.*

## Discussion

Long-term fertilization has reshaped the ecology of the salt marsh plots at Great Sippewissett Marsh (Valiela, 2015 and references therein), but this has primarily been assessed at macro-ecological scales. Increased rates of nitrogen cycling processes in response to nutrient enrichment are commonly observed in both soils and aquatic sediments (Luo et al., 1999; Enwall et al., 2005; Chen et al., 2012; Fierer et al., 2012; McCrackin and Elser, 2012), including in salt marshes (Hamersley and Howes, 2005). Rates of denitrification (Hamersley and Howes, 2005; Peng et al., 2016), N_2_O production (Moseman-Valtierra et al., 2011), and N_2_O consumption (Ji et al., 2015) all increased significantly as a result of nutrient enrichment in Sippewissett Salt Marsh sediments. The Sippewissett Marsh plots have also contributed to our understanding of how increasing N alters denitrification (Valiela and Teal, 1979), coupled nitrification–denitrification (Hamersley and Howes, 2005), and nitrogen retention (Brin et al., 2010). What has received less attention, however, is the effect of prolonged nitrogen enrichment on the structure of the microbial communities that mediate important geochemical transformations. In this study, we use functional gene microarrays and high throughput sequencing to demonstrate that microbial communities mediating N_2_O production and consumption respond to environmental conditions and thus are likely to influence N_2_O flux.

Habitat appears to have little effect on sediment N, water, or oxygen content. N load, however, significantly affects NO_3_^-^ and water content within the sediment, while depth affects oxygen supply. Compared to C and HF plots, XF plots have significantly higher pore water NO_3_^-^ concentrations and lower soil moisture content (**Figure 1**). The relatively low soil moisture in the XF plots is likely due to the increased elevation in the XF relative to C and HF plots (Fox et al., 2012), which results in less frequent flooding and more efficient drainage. The effect of this difference in soil moisture confounds our ability to determine whether the effects on microbial community structure that we observe are a direct effect of the nutrient enrichment or an indirect effect of the nutrient enrichment on other aspects of the geochemistry of the system, such as moisture content. These changes in the physiochemical conditions associated with nutrient enrichment also coincide with increased rates of denitrification previously measured (Valiela and Teal, 1979; Hamersley and Howes, 2005; Peng et al., 2016). These increased rates of nitrogen-cycling processes can arise from a change in the activity of members in a microbial community, while the community composition remains largely unchanged, or as a result of a change in the community composition of the microbes responsible for nitrogen transformations.

Cores taken alongside those analyzed in this study were used to determine N-cycling rates in the sediment (Ji et al., 2015; Peng et al., 2016). These studies showed that long-term fertilization affected both the size and source of the N_2_O flux, as additional N inputs increased the production and consumption rates for N_2_O and shifted the dominant source of N_2_O from ammonia oxidation to denitrification (Ji et al., 2015). While nutrient enrichment did not significantly alter the community composition of ammonia-oxidizing archaea, as evidenced by these microarray results, ammonia-oxidizing bacteria detected in the 16S rRNA in this study were more prevalent in the potentially active community of the control plots, suggesting a role for AOB as an N_2_O source in sites that do not receive N additions. Although ammonia-oxidizing archaea can be more abundant in salt marsh sediments (Moin et al., 2009; Peng et al., 2013), community fingerprinting showed that AOB communities can differ as a result of nutrient enrichment (Peng et al., 2013). Additionally, we observed an increase in the relative abundance of 19 bacterial taxa containing *norB* or *nosZ* genes in response to fertilization (**Table 1**). Taken together, these results suggest that the increased rates of nitrogen-cycling processes result at least partly from a change in the community composition of nitrogen-cycling microbes, as opposed to simply a change in the activity of a static community.

The taxa associated with the 19 archetypes enriched in the fertilized plots likely represent microbes at least partly responsible for the increased rates of N_2_O production and consumption that resulted from fertilization (Ji et al., 2015). These taxa highlight the modularity of denitrification within marsh sediments (Jones et al., 2008; Philippot et al., 2011; Graf et al., 2014; Graves et al., 2016; Roco et al., 2017). In the absence of modularity, abundant *norB* and abundant canonical *nosZ* should derive from the same organism, indicating a complete pathway. In these sediments, the abundant *norB* and *nosZ* probes show no overlap of taxa (**Table 1**). This suggests that major microbial players producing N_2_O are an entirely different suite of organisms than those that consume N_2_O, and thus imply a decoupling between N_2_O production and consumption in salt marsh sediments. This disconnect exists in both C and XF plots, which indicates that the modularity of denitrification in salt marsh sediments exists regardless of N load. When considered with the increased nitrogen cycling rates measured in the Sippewissett plots (Ji et al., 2015; Peng et al., 2016), the modularity we observed may play a key role in determining the magnitude and source of N_2_O fluxes from marsh sediments.

For three of the genes we examined, *norB*, *nosZ*, and atypical *nosZ*1, the archetype with the largest relative abundance was inhibited by fertilization (**Table 2**). However, many relatively less-abundant archetypes were enhanced by fertilization, suggesting that denitrifiers adapted to lower nutrient environments may be outcompeted by taxa that respond positively to nutrient enrichment. Much like macro-organisms, competition is common among micro-organisms (Ghoul and Mitri, 2016 and references therein). Competition for resources can lead to dramatic shifts in community structure. Competition for NH_4_^+^, for example, leads to shifts in abundances of AOA and AOB, in soils, sediments, and the open ocean (Santoro et al., 2010; Beman, 2014; Ouyang et al., 2016). Ecological theory predicts that when nutrients are limiting, the best competitor dominates the community, while in instances where nutrients are not limiting, more taxa are able to succeed (Tilman, 1982), a pattern we observe here within the N-cycling microbial community.

Under control conditions, predictions regarding key producers of N_2_O made by the microarrays were supported by the 16S rRNA data. The archetype for the most dominant member of the *norB* containing taxa corresponds to *Thioalkalivibrio*, which had significantly higher RFR in C plots. *Thioalkalivibrio* was also one of the most abundant taxa in the potentially active community, where it was more abundant in the control than fertilized plots. These data suggest that *Thioalkalivibrio*, along with other AOB identified via 16S rRNA sequencing may be possible sources of N_2_O in control sediments.

In sediments receiving N additions, three of the *norB* archetypes that were enhanced belonged to nitrifying bacteria (*norB14*, *norB15*, and *norB27*) from the genera *Nitrosococcus* and *Nitrosospira*. Of these three, *norB14* and *norB27* had significantly higher RFR in XF plots, which suggests that some of the increased N_2_O production observed in fertilized sediments may be due to increases in nitrifier-denitrification. Nitrifier-denitrification has been extensively studied within the context of microbial bioreactors for waste remediation and nutrient removal (Turk and Mavinic, 1986; Abeling and Seyfried, 1992; Wunderlin et al., 2012). In these systems, nitrifier-denitrification is stimulated by high-N and fluctuating oxygen conditions (Yoo et al., 1999), which are similar to those found in the marsh sediments. Additionally, Zhu et al. (2013) found that nitrifier-denitrification supplied a large portion of the N_2_O flux from soils under low oxygen/high moisture conditions. Nitrifiers identified in the potentially active community were more abundant in wetter C sediments, consistent with the findings from Zhu et al. (2013). *Nitrosococcus* and *Nitrosospira*, however, were rarely observed in 16S rRNA data and did not vary as a result of nutrient enrichment.

Multiple archetypes associated with the atypical *nosZ* sequences also increased significantly as a function of fertilization, indicating that fertilization leads to distinct communities of nitrous oxide scavengers. In fact, 10 atypical *nosZ* archetypes were significantly more abundant in fertilized plots compared to only three archetypes that were more abundant in control sediments. These results provide a potential genetic explanation for increased N_2_O consumption measured in the extra highly fertilized plots (Ji et al., 2015), and they highlight the importance of N_2_O scavengers that act to decrease the elevated N_2_O fluxes associated with high N loads. Atypical *nosZ* have previously been shown to represent a potentially large sink for N_2_O (Jones et al., 2013) and in salt marshes can account for the majority of *nosZ* sequences isolated from sediments (Graves et al., 2016).

Our results highlight the important role nutrient enrichment plays in the structure and functioning of salt marsh ecosystems. These data suggest that of the factors that are believed to control N_2_O flux, in salt marsh sediments, nitrogen supply is the most critical. Nutrient enrichment alters the chemical conditions in the sediment, which in turn alters the community composition of many genes involved in the N cycle, with the exception of archaeal *amoA*. Fertilization also affects the active portion of the microbial community, which is ultimately responsible for the production and consumption of N_2_O, and for all the ecosystem services provided by the marsh. Understanding controls on the balance between production and consumption of N_2_O is critical to determine whether marshes will become a net source of N_2_O under nutrient enrichment. Our results allowed us to identify specific taxa responsible for elevated nitrogen-cycling rates associated with increased N load, thereby identifying which organisms to target for better understanding N_2_O flux. Finally, these data show that in spite of evidence that overall microbial communities in marshes are resistant to change in response to long-term nutrient enrichment (Bowen et al., 2011), the functional capacity of these systems is sensitive to increasing nitrogen supply. In the Great Sippewissett Salt Marsh plots, long-term nutrient enrichment has significantly altered the nitrogen-cycling community within the sediment, with downstream changes in ecosystem function.

## Author Contributions

JA, BW, and JB designed the research, analyzed the data, and wrote the manuscript with contributions from XP, QJ, IC, AJ, and PK. JA, XP, QJ, and PK performed all field work and all geochemical analyses. IC, JA, PK, and JB performed the 16S rRNA and 16S rRNA gene sequencing and the data analysis. JA, AJ, and BW performed all the microarray analyses.

## Conflict of Interest Statement

The authors declare that the research was conducted in the absence of any commercial or financial relationships that could be construed as a potential conflict of interest.
